# Biocompatibility of three-dimensional thermoplastic elastomer implants compared to that of the sternocephalic muscle flap in repairing partial tracheal defects in New Zealand white rabbits (*Oryctolagus cuniculus*)

**DOI:** 10.1590/acb405025

**Published:** 2025-08-08

**Authors:** Marcelo Carrijo da Costa, Ana Carolina Gasques Salgado dos Santos, Ariadne Rein, Gabriel Luiz Montanhim, Gustavo Fernandes, Luis Gustavo Gosuen Gonçalves Dias, Marcella Dall Agnol Leite, Nicolle Pereira Soares, Thiago André Salvitti de Sá Rocha, Marcelo Emilio Beletti, Paola Castro Moraes

**Affiliations:** 1 Universidade Estadual Paulista - Faculdade de Ciências Agrárias e Veterinárias - Departamento de Cirurgia - Jaboticabal (SP) - Brazil.; 2 Universidade Federal de Jataí - Instituto de Ciências Agrárias - Departamento de Cirurgia - Jataí (GO) - Brazil.; 3 Universidade Federal de Uberlândia - Programa de Pós-Graduação em Ciências Veterinárias - Uberlândia (MG) - Brazil.; 4 Universidade Federal de Uberlândia - Instituto de Ciências Biomédicas - Uberlândia (MG) - Brazil.

**Keywords:** Prostheses and Implants, Printing, Three-Dimensional, Rabbits, Histocompatibility

## Abstract

**Purpose:**

To evaluate the effectiveness and biocompatibility of a three-dimensional (3D) thermoplastic elastomer (TPE) prosthesis for repairing partial tracheal defects using 3D printing with fused deposition modelling.

**Methods:**

Thirty-two rabbits underwent partial resection of five tracheal rings and were divided into two groups: the muscular flap group (GFM), which received a sternocephalic muscle flap over the defect; and the TPE group (GTPE), which received a 3D-printed TPE prosthesis. Clinical evaluations were performed at seven, 15, 30, and 60 days postoperatively.

**Results:**

Cough was significantly more frequent in the GFM group (*p* = 0.035). Thermography showed no significant differences in surgical site temperatures between groups or time points. Tracheoscopy revealed more intraluminal secretions in the GTPE group (p = 0.006) and more exuberant tissue formation in the GFM group (*p* = 0.001). Complete epithelialization was observed in the GFM group after 60 days.

**Conclusion:**

The 3D TPE prosthesis demonstrated tissue compatibility and viability as an implant for partial tracheal defects, with minimal respiratory complications during the 60-day evaluation period.

## Introduction

Tracheostomies in humans date back to the second and third centuries. However, the first tracheal resection was only described in the 20th century by Barclay, and in 1990, it was demonstrated the applicability of the technique of resection and tracheal anastomosis^1^-^3^. This technique is most frequently used to treat a segment of tracheal stenosis, which usually occurs because of other causes, such as intubation injuries, tracheal orthyroid tumors, tracheoesophageal fistulas, and trauma^4^,^5^.

Airway reconstruction after resection of stenotic lesions or malignancies is one of the most difficult procedures since the removal of tracheal segments exceeding 50% of the length in adults or one-third in children is generally considered unresectable, due to the excessive strain that prevents end-to-end anastomosis^6^,^7^. Anastomotic complications after the resection procedure and tracheal reconstruction can cause severe stenosis, whether due to the presence of a scar that develops at the suture line or by the formation of granulations in the anastomosis^4^,^8^.

Cartilage, muscle, and skin grafts have been used for the reconstruction of defects after the removal of tracheal areas. Autogenous fabric tissue is ideal for tracheal replacement, but this tissue is available in limited quantities^9^. Anyway, reconstruction requires several complex procedures, and postoperative stenosis may occur because of the formation of granulation tissue^6^.

For the evaluation of the inflammatory process, non-invasive methods such as thermography have been implemented, as several lesions have a significant ability to influence cutaneous blood flow, which affects skin temperature^10^. Another evaluation method is tracheoscopy, which is an important tool for managing patients with difficult-to-treat respiratory diseases. It is generally recommended for the diagnosis of neoplasms and urinary tract infections, respiratory system, in addition to being indicated in cases of haemoptysis^11^.

Synthetic prostheses have been used for reconstructive surgery of tracheal defects, but with the same complications as those previously mentioned^12^. To customize the production of these prostheses, the use of three-dimensional (3D) printing technology has gained prominence in tissue engineering, becoming a highly studied topic since the first publication of the study carried out^13^, which described the application of a scaffold produced from 3D printing in detail^14^.

Modelling by fusion and deposition or fused deposition modelling (FDM) is the most common and cheapest form of additive manufacturing technology, and some models may simultaneously print multiple material types. FDM can be used to produce materials with high-dimensional accuracy and excellent mechanical properties. In medicine, FDM is used to manufacture custom patient-specific devices such as implants, prostheses, anatomical models, and surgical guides. For an effective implant, the biomaterial used for its manufacture must be biocompatible, inert, and durable^15^. Therefore, thermoplastic elastomer (TPE) stands out for their diverse classes and the most varied jobs in the health area when used in instruments, surgical instruments, catheters, coronary stents, and prosthetic limbs^16^.

This study aimed to evaluate the feasibility of using a TPE printed at 86 Shore hardness as a biomedical prosthesis, as well as the biocompatibility of 3D TPE prostheses applied to partial defects in tracheas.

## Methods

### Ethical aspects

Clinical evaluations and surgical procedures were performed at the Veterinary Hospital Governor Laudo Natel. The project was carried out at Universidade Estadual Paulista “Júlio de Mesquita Filho” (UNESP), Jaboticabal campus, SP, Brazil, in partnership with the Universidade Federal de Uberlândia, Uberlândia, MG, Brazil.

This study complied with the ethical principles of the National Council for the Control of Animal Experimentation. The Ethics Committee on Animal Use of the Faculty of Agricultural and Veterinary Sciences, Jaboticabal Campus, approved the project under protocol number 006162/19, in meeting held on May 16, 2019.

### Experimental model

Thirty-two male New Zealand white rabbits (*Oryctolagus cuniculus*), with live weight between 3 and 3.5 kg, physically healthy, from the central vivarium of the Faculty of Veterinary Medicine and Zootechnics, at UNESP, Botucatu campus, SP, Brazil, were used.

The animals were kept in cages with food and water *ad libitum* for two months, a period for adaptation to the environment and management of weight gain before starting the experimental phase.

### Anesthetic protocol

The rabbits were prepared one day before the surgical procedure, with broad trichotomy of the ventral cervical portion. Presurgical fasting was not necessary, because of the particularity of the species^17^.

For the anesthetic protocol, the pre-anesthetic medication used was chlorpromazine hydrochloride, at the dose of 0.5 mg/kg, and morphine, at the dose of 0.5 mg/kg, diluted in the same syringe and administered intramuscularly (IM).

The animals were subjected to a wide trichotomy of the ear region for venous access. For anesthetic induction, propofol was used at the dose of 10 mg/kg, followed by orotracheal intubation with a 3-mm diameter Magill tube with a “cuff,” connected to the anesthesia machine to deliver a total flow of 1 L/min of 100% oxygen through the a valvular anesthetic circuit. The animals were maintained under inhalation anesthesia using isoflurane at the concentration of 3% and diluted in 100% oxygen.

### The three-dimensional prosthesis printing by deposition modelling method

**Figure 1:**
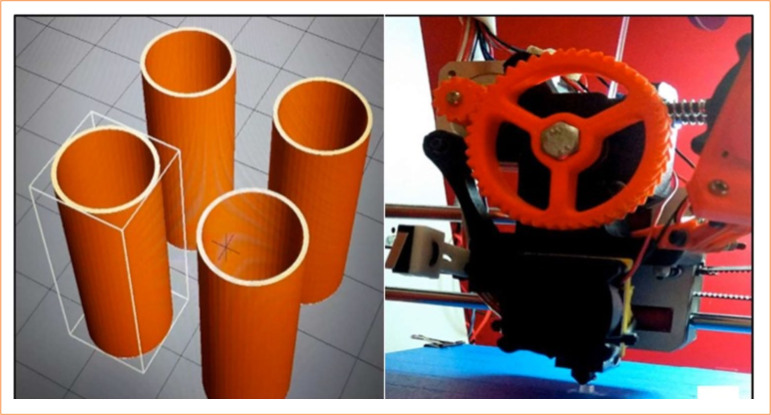
Computational modeling and three-dimensional printing of the thermoplastic elastomer tracheal prosthesis using the fused deposition modeling method.

Source: Elaborated by the authors.

The prostheses were designed in a prototyping computer program, 3D SolidWorks 2016 (Fig. 1a). Afterwards, the file was converted for stereolithography, and printing was executed using modelling by fusion method and deposited in a Prusa printer model I3 (Fig. 1b). The prosthesis was fabricated using a TPE produced by TrueFlex. The dimensions were based on the mean of previous measurements of the mid-tracheal portion of rabbit cadavers, with the following dimensions: length, 15 mm; diameter of cranial circumference, 4 mm; caudal circumference, 5 mm; and wall thickness, 0.5 mm.

**Figure 2:**
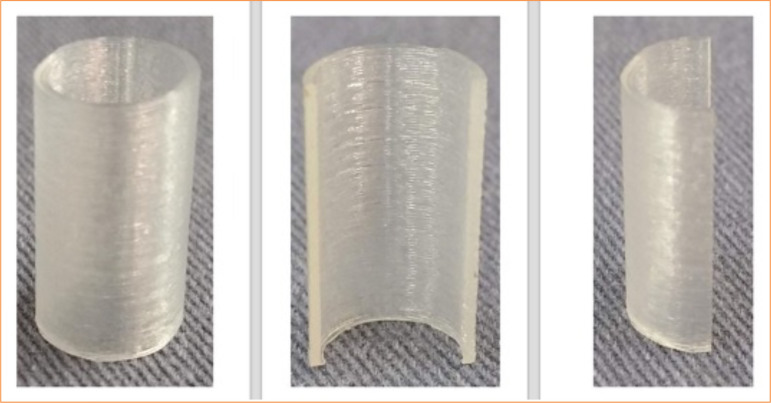
Final aspect of the three-dimensional thermoplastic elastomer tracheal prosthesis after printing and sectioning for partial defect repair.

Source: Elaborated by the authors.

The prototypes were packed in surgical grade paper and autoclaved in a cycle fast at 121°C for 15 min. Figure 2 illustrates the prostheses fabricated using the 3D printer.

### Surgical procedure

After training in the techniques employed, the animals were subjected to the surgical procedure, which was performed by the same surgeon, assistant, and staff.

### Stage common to groups

After definitive antisepsis of the ventral cervical region with chlorhexidine degerming and alcoholic solution and subsequent placement of sterile fields, a longitudinal incision was made in the skin from the cricoid cartilage to the manubrium of the sternum, with subsequent divulsion of the subcutaneous layer and sternohyoid muscles. They were dislocated from the midline, moving them laterally with the aid of Farabeuf retractors. The connective tissues of the trachea were dissected using scissors, Metzenbaum, preserving the lateral portions responsible for vascularization, and recurrent laryngeal nerves. The organs were exposed and isolated through the application of repair sutures, cranial and caudal to the incision site using a needled 3-0 polypropylene suture.

**Figure 3:**
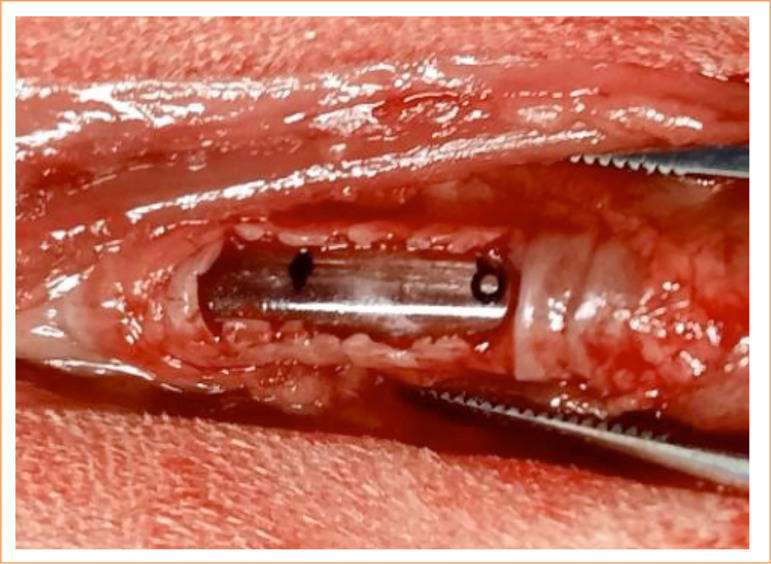
Tracheal ring resection for partial defect in rabbits.

Source: Elaborated by the authors.

Using a number 11 scalpel blade, partial resection was performed on the ventral part of the trachea at the level of the fourth interannular cartilage space up to the eighth space, that is, a defect created by the partial removal of the five tracheal rings (Fig. 3).

### Experimental groups

The 32 animals were divided into two groups of 16 animals each:

- the muscle flap group (GFM), with the removal of the tracheal defect and placement of a flap of the sternocephalic muscle over the defect site;
- the TPE group (GTPE), with tracheal defect removal and 3D TPE prosthesis fixation.

In each group, the animals were subdivided into four groups (n = 4) according to the time of euthanasia (seven, 15, 30, and 60 days).

**Figure 4:**
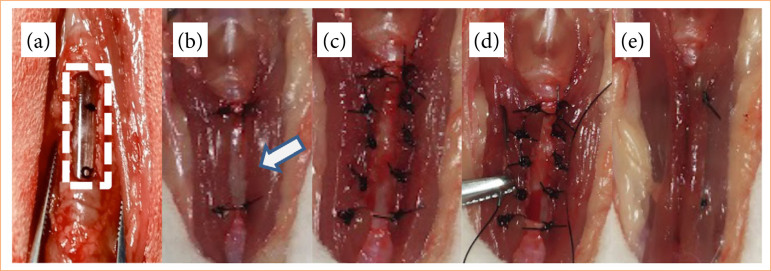
Surgical technique for repairing partial tracheal defect in rabbits using bilateral sternocephalic muscle flap.

Source: Elaborated by the authors.

In the GFM, after creating the tracheal defect, the sternocephalic muscles and previously prepared partial defect were covered by a bilateral advancement flap, in which they were initially fixed using two sutures in separate simple patterns distributed at each end of the defect. In each lateral border of the defect, three sutures were placed in a separate simple pattern in the musculature and subsequent fixation using 4-0 polypropylene. The central region was positioned using a gel-type suture, resulting in approximation and sealing defects in musculature. The cranial and caudal ends of the muscles over the defect were sutured in a Wolf-type pattern (Fig. 4).

In GTPE, after creating a partial tracheal defect and immediately after the lesion, four cardinal repair points were made at the ends of the prosthesis.

**Figure 5:**
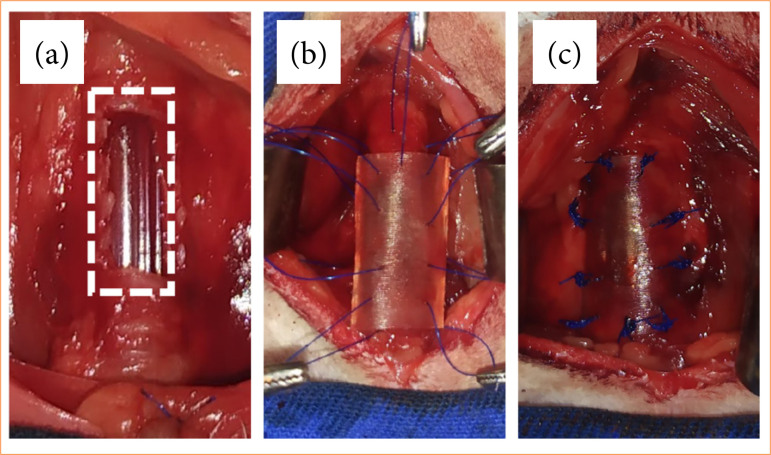
Fixation of thermoplastic elastomer prosthesis in partial tracheal defect in rabbits.

Source: Elaborated by the authors.

Subsequently, sutures were distributed around the entire contact interface area prosthesis/trachea using a 3-0 needled polypropylene thread (Fig. 5).

In all animals, regardless of the group, the subcutaneous tissue was approximated by continuous suture using a 3-0 needled polypropylene thread, and in the skin, a simple pattern separated by the same thread was used.

### Immediate postoperative management

After the surgical procedure, the animals were intubated for 10 min to gradually reduce the percentage of inhalational anesthetics. An endotracheal tube was pulled cranially with the cuff slightly inflated to remove blood clots and secretions from the lumen. Recovery from anesthesia was evaluated using pulse oximetry. After extubating and oximetry (98%), the rabbits were assigned to their respective cages.

Food and water supply followed the routine established in the adaptation phase and were offered soon after awakening from anesthesia.

As a postoperative therapeutic protocol, antimicrobial therapy was instituted with enrofloxacin at the dose of 5 mg/kg administered subcutaneously every 12 h for seven days.

Tramadol hydrochloride was administered as an analgesic at the dose of 4 mg/kg per day, subcutaneously, every 12 h for five days. No drug immunosuppressant was administered to the animals during this process.

### Surgical clinical evaluation

Daily assessment of the breathing pattern was performed to detect possible complications such as dyspnea, cyanosis, breath sounds, cough, and any alterations attributed to the surgical procedure such as oedema, hematoma, emphysema subcutaneous tissue, and the presence of local secretions.

### Thermographic evaluation

To capture the thermographic images, a FLIR camera model was used. The T-300 was adjusted to focus on the distance of 30 cm with automatic focus adjustment. This model had a thermal sensitivity of 0.05° and IR resolution of 320 × 240 (76,800 pixels), in addition to capturing a temperature range varying from -20 to 650°C. All rabbits were shaved 24 h before the surgical procedure and the thermographic evaluation since the hairs also acted as a physical barrier to the emission of IV waves, which may interfer with the results18. One hour before each surgical procedure, animals were placed in a room heated to 20°C. Thermographic evaluation of the tracheal region was performed 30 cm from the cervical region immediately after surgery.

Anesthesia was induced perioperatively and before euthanasia (seven, 15, 30, and 60 days).

### Euthanasia

Each group was subjected to scheduled euthanasia at seven, 15, 30, and 60 days, with intravenous administration of propofol (dose-response), and upon verifying the absence of corneal and pupillary reflexes, 20 mL of intravenous potassium chloride was administered to induce asystole.

### Evaluation by tracheoscopy

**Figure 6:**
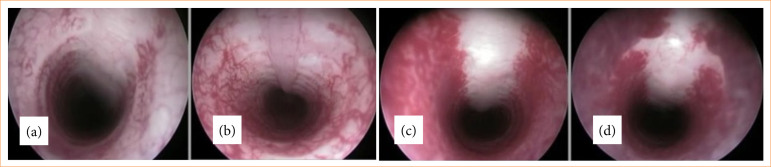
Tracheoscopy of healthy rabbits showing normal tracheal lumen.

Source: Elaborated by the authors.

Tracheoscopies were performed on four rabbits immediately after euthanasia, originating from another experimental work, but by the same team, with origin, rearing, and food management identical to those of the animals used in the present study, without any surgical intervention or previous orotracheal intubation, with the objective to obtain images of the normal trachea of the control group (Fig. 6).

The images captured for the control group supported the principle used by most bronchoscopists who performed the subjective and variable estimation of central airway obstruction in the absence of a clinical practice^19^.

A tracheoscopy was performed immediately after euthanasia. The device used was a 4-mm, 30° rigid endoscope (Karl Storzendoskope, Telecam SL NTSC micro-camera, Hopkins II optics with halogen cold light source 250 Twin and Xenon 100). The endoscope was introduced into the oral cavity of the animal after slight lateral traction of the tongue, which progressed through the oropharyngeal region, until it was possible to visualize the larynx and trachea. The images were captured and stored in an Acer Aspire 5741-5775 notebook.

They were analyzed for the presence of secretions, exuberant tissues, and tracheal stenosis. Stenosis was classified according to the degree of narrowing of the lumen based on a previous study and the criteria established by Freitag et al.^20^, considering 0%: absent, < 25%: discrete, 26-50%: moderate, and > 50%: severe. The presence of secretions and exuberant tissues were categorized as present or absent.

### Macroscopic evaluation

After euthanasia, the site of the partial tracheal defect was surgically assessed and evaluated macroscopically via direct inspection. Comments such as suture dehiscence, seroma, contamination, or subcutaneous emphysema were graduates in absent (-), discreet (+), moderate (++), and intense (+++).

### Histopathological analyses

After euthanasia, the tracheas were dissected and removed until 2 cm cranially to the carina for histopathological analysis. The fragments were kept in vials containing 10% formaldehyde for at least 72 h and sent for the manufacture of the slides. The trachea segments fixed in formaldehyde were dehydrated in an increasing series of alcohols, cleared, and embedded in histological paraffin, and then semi-serial sections were prepared on the microtome (Leica model RM2145) with a 5-µm thickness. Slides were prepared and stained with hematoxylin-eosin (HE). The slides were processed using the Scanscope AT slide scanner and subsequently read using the Aperio software ImageScope version 12.1.

The prosthesis/trachea and flap/trachea interface areas were analyzed using evidence of rejection, dehiscence, and repair, with special attention paid to changes related to replacement by fibrosis, the presence of necrosis in the interface region, granulomas, strictures, ingrowth of epithelium, infection, neovascularization, and collagen proliferation/hypertrophy.

### Criteria for evaluating the biocompatibility of the prosthesis

In biocompatibility, the following parameters were evaluated: type of inflammatory response; type of inflammatory infiltrate; congestion and oedema, identifying the presence of vascular congestion, cellular or interstitial oedema, and fibrosis; and the presence of connective tissue.

### Statistical analysis

Regression diagnosis was performed for data processing to assess whether the data followed a normal distribution.

Kolmogorov-Smirnov and Shapiro-Wilk’s tests were used to determine normal distribution data when *p* > 0.05. The variables with a normal distribution were subjected to parametric tests, t-test and one-way analysis of variance (ANOVA) test. Non-parametric Mann-Whitney and Kruskal-Wallis’ tests were used for variables that did not show a normal distribution. All analyses were performed with 95% confidence intervals.

### Results

### Macroscopic assessment

Macroscopic evaluation of the surgical wound site did not confirm suture dehiscence, seroma, subcutaneous contamination, or emphysema in any of the groups and periods evaluated.

Necropsy was performed on animals that died before the period of euthanasia, with a focus on the study area. In animals from the GFM group, a large amount of serous secretion was observed without an increase in local volume, suture dehiscence, or stenosis in the tracheal lumen for 60 days. However, no serous secretion, local swelling, implant suture dehiscence, or severe reduction of the tracheal lumen were observed in those in the GTPE group.

### Surgical clinical evaluation

Tracheal stridor, cough, and dyspnea were the clinical signs evaluated. In the GFM group, seven animals (43.75%) showed clinical signs, with greater occurrence within 15 days (two animals, 50%). The GTPE group presented only one animal (6%) with one of the clinical signs that occurred during the 30-day study period. Table 1 presents the data observed in the animals and their respective periods.

**Table 1:** Distribution of clinical findings of rabbits subjected to repair of a partial defect of the trachea with a flap of the sternocephalic muscle and a three-dimensional (3D) thermoplastic elastomer prosthesis. Jaboticabal, SP, Brazil, 2022.

	7 days (%)		15 days (%)		30 days (%)		60 days (%)		Total Mann-Whitney’s U test (average of ranks)	
Groups evaluated	GFM	GTPE	GFM	GTPE	GFM	GTPE	GFM	GTPE	GFM	GTPE
Stridor	25	-	50	-	50	25	25	-	18.50	14.50
Cough	-	-	50	-	25	-	25	-	18.50*	14.50*
Dyspnea	-	-	-	-	-	-	25	-	17.00	16.00

GFM: muscle flap group; GTPE: thermoplastic elastomer prosthesis group; -: absent; *p < 0.001.

Among the 32 New Zealand rabbits subjected to the procedure, two died (12%), both in 60 days, one each in the GFM and GTPE groups. The animals in the GFM group had severe dyspnea, cyanosis, position orthopnea, and rapid progression to death. However, animals in the GTPE group did not show clear clinical signs before death.

No statistically significant differences were observed between the GFM and GTPE groups for stridor and dyspnea (Mann-Whitney’s test, *p* > 0.05), regardless of the analyzed period. However, there was a statistically significant difference in the clinical signs of cough, with a greater occurrence in the GFM group than that in the GTPE group (Mann-Whitney’s U test, *p* = 0.035).

### Evaluation of thermographic images

**Figure 7:**
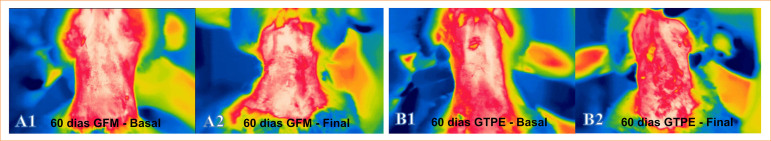
Thermography of the cervical region in rabbits undergoing partial tracheal repair in GFM and GTPE.

GFM: muscle flap group; GTPE: thermoplastic elastomer prosthesis group. Source: Elaborated by the authors.

The thermographic images referring to the animals belonging to the GFM and GTPE groups were captured using the FLIR T-300 camera, in two moments, before the procedure (baseline) and before euthanasia, at seven, 15, 30, and 60 days. FLIR Tools software was used to measure the minimum, maximum, and average temperatures (Fig. 7). The analyzed data are presented in Table 2.

**Table 2:** General averages of temperature data obtained by FLIR Tools software from the images captured by the FLIR T-300 camera, of the ventral cervical region of rabbits, subjected to repair of partial trachea defect with muscle flap and three-dimensional thermoplastic elastomer prosthesis implant, in the evaluated periods of seven, 15, 30 and 60 days. Jaboticabal, SP, Brazil, 2022.

		N	Average	Standard deviation	Mean standard error	Meaningfulness
Temperature	GFM	15	35.9467	0.69294	0.17892	0.764
	GTPE	15	35.5300	0.72624	0.18752	

GFM: muscle flap group; GTPE: thermoplastic elastomer prosthesis group.

A one-way ANOVA test was used, and there were no statistically significant differences between the temperatures of the GFM and GTPE groups or within the groups during the evaluated periods (F (3.26) = 0.0386, (*p* = 0.764).

### Tracheoscopy assessment

At tracheoscopy, yellowish mucus secretion was observed in 46% of the animals in the GFM group, with a greater occurrence for 15 days (75%); in the GTPE group, 93% of the animals presented an occurrence of secretion. The fabric visualization exuberance only occurred in the GFM group, with a greater occurrence in the seven days (100%). Different degrees of stenosis were observed in both groups, in the GFM group, 30 days was the most frequent period (50%), while the GTPE had a higher occurrence for seven days (50%). The distribution of findings in tracheoscopy tests is shown in Table 3.

**Table 3:** Distribution of tracheoscopy findings of rabbits in the evaluated periods of seven, 15, 30, and 60 days, in the groups submitted to partial tracheal repair with sternocephalic muscle flap (SFM), and in the group with partial tracheal repair using three-dimensional thermoplastic elastomer implant. Jaboticabal, SP, Brazil, 2022.

	7 days (%)		15 days (%)		30 days (%)		60 days (%)		Total Mann-Whitney’s U test (average of ranks)	
Groups evaluated	GFM	GTPE	GFM	GTPE	GFM	GTPE	GFM	GTPE	GFM	GTPE
Secretion	25	100	75	100	50	75	33	100	11.10*	19.90*
Stenosis light	-	25	25	-	-	-	-	-		
Moderate	-	-	-	-	50	25	-	-	15.60	15.40
accentuated	-	25	-	-	-	-	-	-		
Tissue lush	100	-	25	-	50	-	33	-	18.50*	12.50*

GFM: muscle flap group; GTPE: thermoplastic elastomer prosthesis group; -: absent; * *p* < 0.05; stenosis: 0%, absent; < 25%, mild, 26-50%: moderate; > 50%, severe.

**Figure 8:**
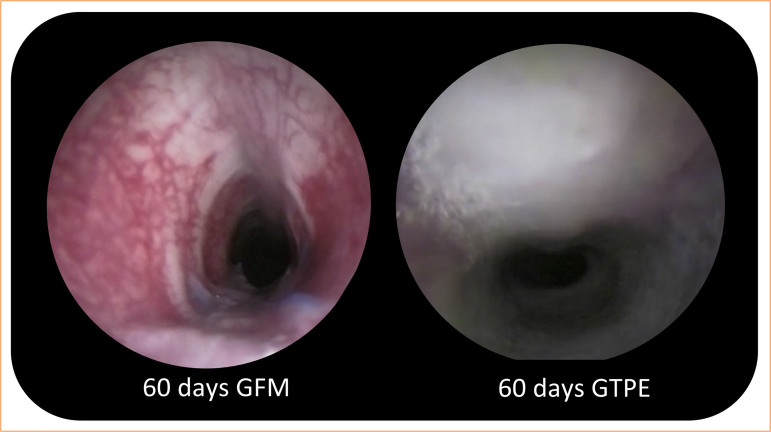
Tracheoscopy at 60 days in GFM and GTPE groups showing lumen patency and secretion accumulation.

GFM: muscle flap group; GTPE: thermoplastic elastomer prosthesis group. Source: Elaborated by the authors.

Secretion visualization was statistically significant in the GTPE group (Mann-Whitney’s test, *p* = 0.006) compared to that in the GFM group. Visualization of exuberant tissue was statistically significant in the GFM group (Mann-Whitney’s test, *p* = 0.001) compared to that in the GTPE group. The occurrence of stenosis showed no statistical difference in the periods and groups evaluated (p = 0.95). The tracheoscopy images of the GFM and GTPE groups at 60 days are illustrated in Fig. 8.

### Histopathological analyses

#### The degree of inflammatory infiltrate (polymorphonuclear cells or mononuclear cells) and neovascularization

The interface areas between the trachea and the flap muscles were evaluated in the GFM group. The formation of new epithelium was observed in the region of the tracheal defect in animals at 30 (75%) and 60 days (100%), completely covering the lumen in the muscle flap region.

**Figure 9:**
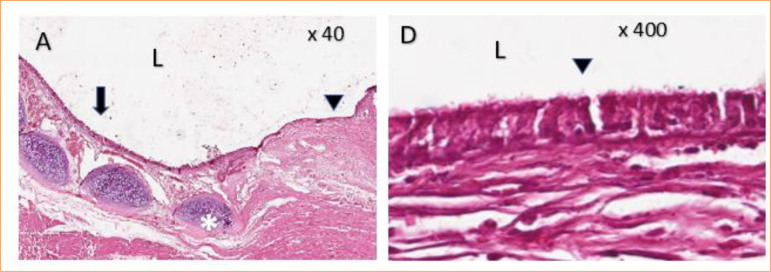
Histology of the interface between trachea and muscle flap after 30 days of repair in rabbits.

Source: Elaborated by the authors.

Microscopy revealed complete respiratory mucosa newly formed by pseudostratified epithelium, goblet cells, and cilia. The lesion area was composed of a discrete proliferation of immature connective tissue formed by reactive fibroblasts and discrete focal mononuclear inflammatory infiltrates, in addition to areas with mild neovascularization and passive hyperemia. There was no intense necrosis, with a continuous solution in place, showing integration between the last tracheal ring and the sternocephalic muscle (Fig. 9).

TPE prostheses were removed from the GTPE group due to the impossibility of performing histological section. The area surrounding the prosthesis interface was evaluated in 3D of the TPE and trachea. The infiltration of polymorphonuclear cells (25%) was noted only on seven days, but the difference was not statistically significant (*p* = 0.47) compared to those associated with the other periods in the GTPE group. The predominant infiltrates in evaluated periods (15, 30, and 60 days) were mononuclear inflammatory cells. However, there was no statistical difference in the type or intensity of infiltrate inflammation or neovascularization within the periods evaluated in the GTPE group.

Under a microscope, the lesion area surrounding the 3D prosthetic interface was identified as the TPE and trachea, with chondrocyte hypertrophy and tissue proliferation connective tissue consisting of reactive fibroblasts and mononuclear inflammatory infiltrates with a moderate focus, in addition to areas with moderate and passive hyperemia.

**Figure 10:**
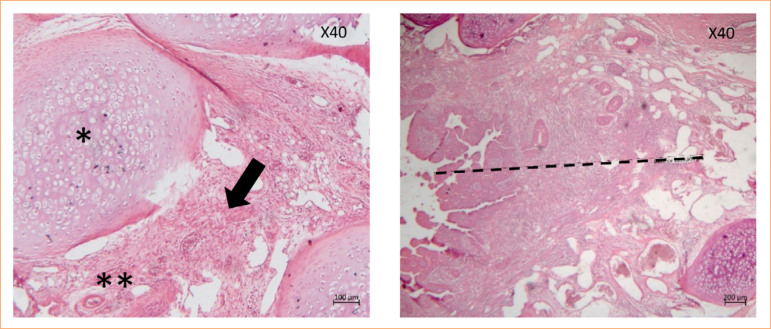
Histology of the interface between three-dimensional thermoplastic elastomer prosthesis and trachea after 60 days of repair in rabbits.

Source: Elaborated by the authors.

No areas of necrosis, edema, or discontinuity were observed at the implantation site of the 3D TPE prosthesis at any of the evaluated time points (Fig. 10).

## Discussion

Tracheal tissue is largely composed of rings of hyaline cartilage and the respiratory mucosa on the luminal surface. Cartilage can sustain tracheal patency during breathing and has sufficient elasticity to withstand changes in pressure during coughing^12^.

The respiratory signs of cough, stridor, and dyspnea are frequently noted and often evaluated in studies with the use of prostheses or tracheal repairs^7^,^21^,^22^. In a previous study^23^, with the implantation of an artificial trachea made by 3D printing together with autologous epithelial cells, most rabbits had abnormal breath sounds, such as stridor, shortly after the implantation procedure, but it improved in 12 months. However, when submitting rabbits for implantation of a polycaprolactone tracheomimetic prosthesis, other authors did not observe any disorders^21^. In the present study, there were no respiratory signs in the GTPE group at 60 days.

Unlike the GFM group, the GTPE group presented at least one respiratory sign and histopathological findings of hyperemia throughout the observation period, which may indicate venous congestion. The exuberant tissue visualized on tracheoscopy may suggest the cause of these manifestations. Furthermore, the use of an epigastric myofascial flap was reported to repair a defective trachea in swine^24^, in which venous congestion was observed, and bronchoscopy revealed the presence of yellowish secretion over the lesion site, as well as in the tracheoscopic evaluation of the GFM group in the present study, together with the histological and imaging findings of the muscle flap group might have culminated in the respiratory clinical signs observed predominantly in this group.

Thermography was used to monitor surgical wounds, and in previous studies^25^-^27^, it proved to be effective as an aiding method in the diagnosis of infections, wound inflammation, and surgical beds. There was no statistically significant difference between the GFM and GTPE groups when the thermographic findings were analyzed, and the temperatures were recorded immediately before and after the surgical procedure.

This fact added to the data obtained in the histopathology showing the formation of respiratory mucosa in the GFM group from 30 days after surgery, suggesting a reduction of the process inflammation with the evolution of the tissue regeneration process. This hypothesis is reinforced by Park et al.^23^, who observed the presence of an inflammatory reaction in the control group of tracheal 3D bioprosthesis implantation, which resulted in the inhibition of epithelial cell regeneration with no formation of the respiratory mucosa.

However, in the experimental group, epithelial cells differentiated into ciliates and were observed in all rabbits 90 days after the surgical procedure, with a structure similar to that of a normal trachea, and this group showed minimal inflammatory response.

For the GTPE group, the non-significant variation in temperature might indicate a non-maintenance of the acute inflammatory response to the 3D prosthesis material of the TPE; these data together might indicate good tissue acceptance to the 3D TPE prosthesis. An acute inflammatory response pattern characterized by polymorphonuclear cells was observed only seven days after the surgical procedure^28^, whereas at the other evaluated time points, the inflammatory pattern was moderate and predominantly composed of mononuclear cells. Thermography is used to accurately identify the acute inflammatory process in paranasal sinuses^29^, and after surgical extraction of molars in humans30, demonstrating the high precision of this method.

The tracheal lumen was evaluated by examining the tracheoscopy, after each moment of euthanasia. Luminal reductions observed for up to 30 days were not maintained in the final analysis at 60 days in any of the evaluated groups. In a previous study, a simple lesion of the tracheal submucosa could induce severe stenosis at a high mortality rate and that maturation of the wound scar is definitive 28 days after induction in rabbits^31^. In the present study, changes in lumen reduction suggested that it was not progressive. Also noteworthy is the absence of serious collapses at the tracheoscopy examination. In a study carried out by Dang et al.^32^, the occurrence of severe tracheal collapse in rabbits subjected to tracheal transplantation decellularized and evolved to death in all animals in the experimental group.

The presence of secretions in prostheses or reconstructive procedures of the tracheal lumen has been observed in several studies^24^,^33^-^35^.

The site of the prosthesis or reconstruction allows stasis of the mucus produced along the trachea owing to the absence of the ciliary system responsible for mucus expectoration^36^. However, it becomes worrisome when there is the formation of retention that blocks the passage of air, with the need for intervention using auxiliary methods, such as bronchoscopy^37^. In both groups, there was no need for the intervention to remove the mucous plugs, which would make it impossible for air to pass.

The presence of reactive fibroblasts and repair connective tissue surrounding the area of the 3D TPE prosthesis, observed histologically, is an unwanted result arising from primary healing^38^.

A study in rabbits observed that the presence of connective tissue at the injury site was a complication in rabbits, because this tissue proliferated into the lumen trachea and led to stenosis, visualized 60 days after the initial procedure and without the regression of the formation^32^. In the present study, after 60 days of evaluation, the fibrotic connective tissue was confined to the areas surrounding the 3D TPE prosthesis without impeding the flow of air because it did not invade the lumen or cause significant external compression.

The emergence of 3D printing technology has resulted in numerous possibilities for tissue engineering^39^. In this study, the tracheal prosthesis, which was designed and printed in 3D, allowed the anatomical dimensions to correspond to those of a healthy rabbit trachea, demonstrating the effectiveness of personalization and adaptation of 3D prostheses to the site of interest^40^. The use of 3D polyurethane partial dentures obtained results similar to these of the present study, with maintenance of tracheal patency, good accommodation of the prosthesis to the wound bed, and absence of signs of contamination.

These results suggest that the TPE used for fabricating the partial tracheal prosthesis was tissue compatible. In addition, it acts as a solid functional support both in the initial-, seven- and 15-day stages, and in the final evaluation period at 30 and 60 days after the repair surgery.

To the best of our knowledge, this study is the first to highlight the defective partial trachea correction using TPE TrueFlex in an animal model.

## Conclusion

The 3D TPE prosthesis proved to be viable for implantation in partial defects of the trachea with minimal respiratory complications over 60 days and no fistulation, dehiscence, or stenosis, contraindicating the use of this technique. Tracheoscopy findings and the observed inflammatory process corroborated the results described in other studies with biocompatible materials, without need for immunosuppressant use to prevent rejection of the prosthesis.

## Data Availability

All data sets were generated or analyzed in the current study.
